# Prévalence du Diabète et Facteurs de Risque Cardiovasculaire Associés dans une Population Rurale au Burkina Faso

**DOI:** 10.48327/B1J8-7K63

**Published:** 2021-02-12

**Authors:** L. Séré, H. Tiéno, D. Yanogo, S. Traoré, Y. Nagabila, D.D. Ouédraogo, Y.J. Drabo

**Affiliations:** 1 Centre hospitalier universitaire de Tengandogo, Ouagadougou, Burkina Faso; 2 Centre hospitalier universitaire de Bogodogo, Ouagadougou, Burkina Faso; 3 Centre hospitalier universitaire Yalgado Ouédraogo, Ouagadougou, Burkina Faso

**Keywords:** Diabète, Facteurs de risque, Dépistage, Milieu rural, Gourcy, Boussou, Lèba, Tougo, Bassi, Zondoma, Burkina Faso, Afrique subsaharienne, Diabetes, Risk factors, Screening, Rural area, Gourcy, Boussou, Lèba, Tougo, Bassi, Zondoma, Burkina Faso, Sub-Saharan Africa

## Abstract

**Objectifs:**

Le milieu rural est confronté de nos jours à des changements du mode de vie qui jouent un rôle important dans l'augmentation de la prévalence du diabète sucré et des autres facteurs de risque cardio-vasculaire. Le but de cette enquête était de déterminer la prévalence et les facteurs de risque associés au diabète sucré dans une population rurale du Burkina Faso.

**Méthodologie:**

Nous avons récolté au cours d'une campagne de dépistage volontaire du diabète des variables épidémio-cliniques et mesuré la glycémie capillaire de tous les sujets à jeun de 16 ans et plus dans les communes de la province du Zondoma.

**Résultats:**

Neuf-cent-soixante-dix personnes d'un âge moyen de 49,06 ± 16,97 ans se sont soumises volontairement au test de dépistage. La majorité des personnes avait un âge compris entre 40 ans et 65 ans (48,5%) et 57,5% étaient des femmes. La prévalence du diabète sucré était de 5,7% et 9% des sujets avaient une glycémie entre 1,10 g/l et 1,26 g/l (hyperglycémie modérée à jeun). Un antécédent familial de diabète a été retrouvé dans 4,3% des cas. Pour les autres facteurs de risques cardio-vasculaires, 24,3% de la population était en surcharge pondérale et 23,6% avait une hypertension artérielle. L'âge (p = 0,001), la profession (p = 0,015) et l'IMC (p = 0,036) étaient significativement associés au diabète.

**Conclusion:**

Le diabète sucré est présent en milieu rural au Burkina Faso avec une proportion non négligeable de pré-diabète. L'âge, la profession et l'IMC sont les principaux facteurs associés.

## Abstract (en)

**Objective:**

Rural areas are currently facing lifestyle changes which greatly contribute to an increase in the prevalence of diabetes and cardiovascular diseases. The aim of this study was to estimate the prevalence of Diabetes and Diabetes-related cardiovascular diseases in a rural population in Burkina Faso.

**Methodology:**

We collected demographic and clinical data, and we also performed capillary glucose testing for all the participants aged 16 years and above in five administrative communities in the province of Zandoma.

**Results:**

In all, 970 individuals, with a mean age of 49.06 years (SD = 16.97), volunteered to participate in the screening. Most of them were aged between 40 and 65 years old (48.5%) and 57.5% were females. The prevalence of diabetes was 5.7%, and 9% of participants had an FPG level between 1.10 g/l and 1.26 g/l. A family history of diabetes was found in 4.3% of the cases. Looking at the other cardiovascular risk factors, we found 24.3% of the participant to be overweight or obese, 23.6% had High Blood Pressure. Age (p = 0.001), Employment Status (p = 0.015), Body Mass Index (p = 0.036) were significantly associated with diabetes.

**Conclusion:**

Diabetes is prevalent in rural areas in Burkina Faso, along with a significant proportion of prediabetes cases. Age, Employment Status, and BMI are the principal associated factors.

## Introduction

La prévalence du diabète au Burkina Faso est de 4,9% au plan national et de 13,9% en milieu urbain [14]. À l'instar du milieu urbain, le monde rural est confronté aux changements du mode de vie.

Le but de notre étude était de déterminer la prévalence du diabète et d'identifier les facteurs de risque cardiovasculaires associés dans une population rurale du Burkina Faso.

## Patients et Méthode

**Fig. 1:**
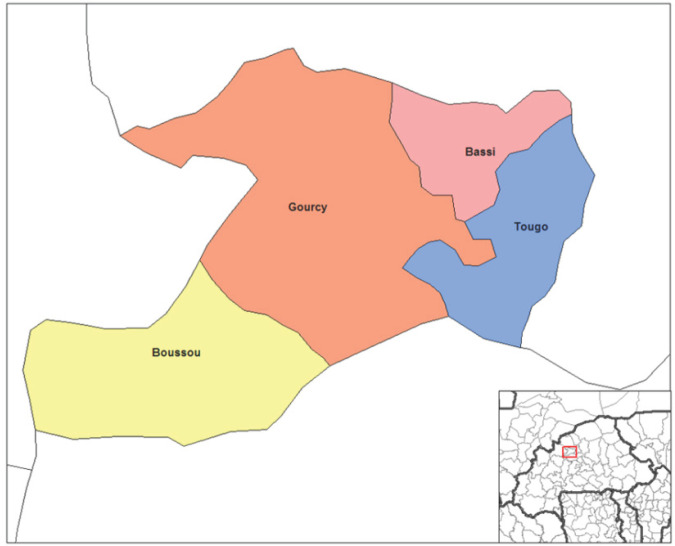
Province du Zondoma Zondoma province

Il s'agit d'une étude transversale ayant concerné les sujets âgés de 16 ans et plus reçus lors d'une campagne de dépistage du diabète organisé dans les centres de santé des cinq communes de la province du Zondoma dans la région du nord du Burkina Faso (Fig. 1). La province du Zondoma est une des quatre provinces de la région du nord du Burkina Faso. Elle a une population de 190869 habitants qui vivent sur une superficie de 1993,88 km^2^ [11]. La population est composée en majorité d'agriculteurs et d'éleveurs. Les principales cultures vivrières sont les céréales (maïs, mil, riz…) et les légumineuses (haricot, pois de terre…) et la pomme de terre. Le cheptel est composé par les bovins et les petits ruminants.

Le dépistage a eu lieu du 24 août au 29 août 2015. En pratique, nous avons organisé une journée de dépistage successivement dans les communes que sont Gourcy, Boussou, Lèba, Tougo et Bassi. Ces journées de dépistage qui sont les premières du genre dans la province, avaient été précédées de deux semaines de campagnes d'informations pour une mobilisation sociale à l'aide de spots publicitaires par la radiodiffusion locale privée « SAVANE FM » et le journal régional « La Voix du Nord ». Les informations étaient données en français et traduites en mooré qui est la langue la plus parlée dans la province.

La participation à la campagne était volontaire et nous avons inclus tous les sujets tout-venant répondant aux critères d'âge et s'étant présentés à jeun sur le site du dépistage entre 7h et 12h du matin.

Les femmes enceintes et les personnes qui n'étaient pas à jeun ont été exclues de l'étude.

Des informations relatives au sexe, à l'âge, à la profession, au statut matrimonial, aux antécédents personnels et familiaux de diabète, ont été collectées lors d'entretiens individuels.

L'indice de masse corporelle (IMC) et le périmètre abdominal ont été utilisés pour apprécier cliniquement le statut pondéral et l'état nutritionnel des sujets.

Les critères de l'OMS ont été utilisés pour catégoriser le statut pondéral des participants. L'obésité abdominale a été définie selon la National Cholesterol Education Program-Adult Treatement Panel III (NCEP- ATP III) par un tour de taille ≥ 102 cm chez l'homme et ≥ 88 cm chez la femme [7].

Deux prises de tension artérielle ont été effectuées, chez chaque personne, assise et au repos depuis au moins 10 minutes, puis la moyenne a été retenue. Le patient était considéré comme hypertendu s'il était déjà traité pour une HTA ou si sa tension artérielle systolique était supérieure ou égale à 140 mm Hg et/ou sa tension artérielle diastolique supérieure ou égale à 90 mm Hg quel soit le genre.

Une glycémie à jeun supérieure ou égale à 1,26 g/l (≥ 7 mmol/l) après deux contrôles à 48 heures d'intervalle définissait le diabète. La glycémie était mesurée à l'aide d'un appareil d'autocontrôle glycémique utilisant des bandelettes réactives de la marque Contours TS.

Les sujets étaient classés en trois groupes selon les normes diagnostiques internationales [15]:

- les patients non diabétiques et dont la glycémie capillaire à jeun était inférieure à 1,10 g/l;
- les patients ayant une hyperglycémie modérée à jeun, soit une glycémie capillaire comprise entre 1,10 g/l et 1,26 g/l;
- les patients diabétiques, soit parce qu'ils avaient déjà auparavant été diagnostiqués diabétiques, soit parce qu'ils avaient une glycémie capillaire à jeun supérieure à 1,26 g/l avec les mêmes résultats au contrôle.

Les données ont été analysées à l'aide du logiciel SPSS Version 20.0. Le test t de Student a été utilisé pour comparer les moyennes, le seuil de signification retenu était de 5%.

## Résultats

Nous avons inclus 970 personnes dont un effectif de 310 personnes à Gourcy (32%), 259 personnes à Tougo (26,7%), 157 personnes à Bassi (16,2%), 140 personnes à Boussou (14,4%) et 104 personnes à Léba (10,7%). L'échantillon était composé de 558 femmes (57,52%) et de 412 (42,48%) hommes, soit un sex-ratio (H/F) de 0,7. L'âge moyen était de 49,06 ± 16,97 ans. La tranche d'âge de 40 ans à 65 ans représentait 48,5% et les sujets de moins de 40 ans 33,7%. La majorité des sujets n'était pas scolarisée (75,4%) et les paysans étaient les plus nombreux (94,5%).

Sur le plan des antécédents personnels, 16 personnes (1,6%) se savaient déjà diabétiques et 142 (14,6%) hypertendus. Les antécédents de diabète concernaient 1,3% des femmes et 2,2% des hommes (p = 0,261). Six personnes (0,6%) étaient à la fois diabétiques et hypertendues.

L'IMC moyen était de 21,10 ± 4,41 kg/m2. Le surpoids était retrouvé chez 153 sujets (18,5%) et l'obésité chez 56 sujets (5,8%). L'obésité androïde touchait 289 (29,8%) des personnes. Les caractéristiques sociodémographiques et les facteurs cardio-vasculaires sont résumés dans le tableau I.

**Tableau I:** Caractéristiques sociodémographiques et facteurs de risque cardio-vasculaire de la population étudiée Socio-demographic characteristics and cardiovascular risk factors of the population under study

Variables			Sexe		p
		Effectifs n (%)	Masculin n (%)	Féminin n (%)	
**Tranche d'âge**					
	< 40 ans	326 (33,7)	134 (32,5)	192 (34,4)	0,539
	40-60 ans	470 (48,5)	196 (47,6)	274 (49,1)	0,637
	> 65 ans	174 (17,9)	82 (19,9)	92 (16,5)	0,171
**Niveau de scolarisation**					
	non scolarisé	731 (75,4)	269 (65,3)	462 (82,8)	0,000
	scolarisé	239 (24,6)	143 (34,7)	96 (17,2)	0,000
**Profession**					
	paysans	917 (94,5)	375 (91)	542 (97,1)	0,000
	non paysans (fonctionnaires en activité et retraités, commerçants)	53 (5,5)	37 (9)	16 (2,9)	
**ATCD médicaux**					
	diabète	16 (1,6)	9 (2,2)	7 (1,3)	0,261
	HTA	142 (14,6)	72 (17,5)	70 (12,5)	0,032
**Consommation d'alcool**		153 (15,8)	78 (18,9)	75 (13,4)	0,020
**IMC (kg/m2)**					
	< 18,5	194 (20)	87 (21,1)	107 (19,2)	0,455
	18,5-24,99	567 (58,5)	268 (65)	299 (53,6)	000
	25-29,9	153 (15,8)	48 (11,7)	105 (18,8)	0,002
	≥ 30	56 (5,8)	9 (2,2)	47 (8,4)	000
**Obésité abdominale**		289 (29,8)	25 (6,1)	264 (47,3)	000
**Hypertension artérielle**		229 (23,6)	95 (23,1)	134 (24)	0,729

Nous avons dépisté 39 cas (4,08%) de diabète, ce qui porte la prévalence globale du diabète dans l'ensemble de cette population à 5,7%. Soixante-dix-neuf sujets (9%) répondaient à la définition de l'hyperglycémie modérée à jeun (tableau II).

**Tableau II:** Prévalence du diabète et de l'hyperglycémie modérée à jeun Prevalence of diabetes and moderate fasting hyperglycemia

Variables	Effectifs	Sexe		p
	n (%)	Masculin n (%)	Féminin n (%)	
diabétiques connus	16 (1,6)	9 (2,2)	7 (1,3)	0,261
diabétiques découverts	39 (4,08)	16 (3,9)	23 (4,12)	0,303
total des diabétiques	55 (5,7)	25 (6,1)	30 (5,4)	0,646
hyperglycémie modérée à jeun	79 (8,1)	22 (5,3)	57 (10,2)	0,02

**Tableau III:** Comparaison des facteurs de risque selon l'existence ou non de diabète Comparison of selected factors according to the presence or absence of diabetes

Facteurs		Effectifs	Diabète (%)	p
**Tranche d'âge**				
	< 40 ans	326	7 (2,1)	0,001
	> 40 ans	644	48 (7,5)	
**Sexe**				
	masculin	412	25 (6,1)	0,645
	féminin	558	30 (5,4)	
**Niveau de scolarisation**				
	non scolarisé	731	38 (5,2)	0,267
	scolarisé	239	17 (7,1)	
**Profession**				
	paysans	917	48 (5,2)	0,015
	non paysans	53	7 (13,2)	
**Situation maritale**				
	vivant en couple	809	39 (4,8)	0,89
	ne vivant pas en couple	161	8 (5)	
**IMC (kg/m2)**				
	IMC< 25	762	37 (4,9)	0,036
	IMC≥ 25	208	18 (8,7)	
**Hypertension artérielle**				
	oui	229	12 (5,2)	0,942
	non	741	43 (5,8)	

Parmi les nouveaux diabétiques, 15 (38,5%) étaient en surpoids ou obèses et 34 (87,2%) avaient 40 ans et plus. De façon globale, 31,9% des diabétiques avaient plus de 65 ans. Pour ce qui concerne les sujets ayant une hyperglycémie modérée à jeun, 25 (31,6%) étaient en surcharge pondérale et 54 (68,4%) avaient au moins 40 ans. Treize personnes (6,5%) avaient à la fois une hyperglycémie, une hypertension artérielle et un surpoids ou une obésité.

En analyse bi-variée, les sujets diabétiques étaient plus souvent âgés, non paysans (fonctionnaires en activité et retraités et commerçants) et en surcharge pondérale (tableau III).

La prévalence de l'HTA n'était pas plus élevée chez les diabétiques (19,1%) que chez les non diabétiques (14,4%) (p = 0,942).

## Discussion

Neuf cent soixante-dix personnes ont participé volontairement à notre étude. Cet échantillon malgré sa petite taille par rapport à la population générale des cinq communes a permis de mettre en exergue l'existence du diabète et d'autres facteurs de risque cardiovasculaire associés dans ce milieu rural.

Cette étude donne une estimation de la prévalence de diabète sucré en milieu rural au Burkina Faso, malgré un échantillon non représentatif. Aussi, le volontariat fait que les sujets ayant au moins un facteur de risque cardiovasculaire comme la surcharge pondérale ou hypertension artérielle sont plus sensibles à des messages appelant à un dépistage d'autres facteurs de risque. Ce qui peut conduire à une surestimation de la prévalence. En outre, il aurait fallu faire un échantillonnage en grappe dans les villages que nos ressources limitées n'ont pas permis de réaliser. La modernisation des pratiques culturales et de déplacement réduisant le niveau d'activité physique, associées à de nouvelles habitudes alimentaires en faveur du sucre, du salé et du gras, vont probablement contribuer à faire progresser le diabète et d'autres facteurs de risque cardiovasculaire comme l'hypertension artérielle et l'obésité en milieu rural dans les pays en voie de développement comme le Burkina Faso.

Malgré un IMC moyen peu élevé dans notre population comme chez la plupart des populations sahéliennes [14], la tendance au surpoids et à l'obésité androïde sont à noter. En effet, environ 20% des sujets étaient en surpoids, 5,8% présentaient une obésité, un cas sur quatre étant une obésité androïde. Cette surcharge pondérale était surtout le fait des femmes (p= 0,000), confortant l'observation déjà faite au Burkina Faso au plan national par l'enquête STEPS 2013 et des études en milieu urbain [10, 12, 14].

La prévalence de l'HTA dans l'ensemble de notre population était de 23,6%. Cette prévalence est supérieure à la prévalence nationale qui est de 17,6% [14]. Par contre, une étude réalisée dans la ville de Ouagadougou avait retrouvé une prévalence plus élevée en milieu urbain (30,4%) [10].

Notre étude a montré que 5,7% de la population rurale étudiée était diabétique. Cette prévalence est légèrement supérieure à la prévalence nationale (4,9%) et inférieure à la prévalence urbaine (13,9%) [14]. Ceci témoigne que la prévalence du diabète retrouvée est non négligeable. Dans d'autres zones rurales d'Afrique sub-saharienne, la prévalence du diabète varie de 7,9% au Tchad à 4% en Guinée et à 2,3% au Mali [1, 4, 8].

La plupart des diabétiques avaient au moins 40 ans. C'est la catégorie d'âge habituelle de prédilection du diabète de type 2 [3, 5, 6, 13,]. Ces cas de diabète se recrutaient plus dans les professions autres que les paysans regroupant les fonctionnaires en activité et retraités et les commerçants qui ont une activité physique réduite (p = 0,015). La majorité des patients atteints de diabète était également en surcharge pondérale (p = 0,036), un facteur prédisposant au diabète de type 2 par insulino-résistance.

Contrairement à la prévalence de l'HTA qui est habituellement élevée chez le diabétique de type 2 [6, 10, 11], la différence n'était pas significative dans notre étude (p = 0,9), les sujets diabétiques n'étant pas plus hypertendus que les autres. Par ailleurs, un syndrome métabolique dans cette population se manifeste par la fréquence (6,5%) des personnes ayant à la fois une hyperglycémie (modérée ou diabétique) et une hypertension artérielle.

La prévalence de diabète méconnu était de 4,08%, soit plus de deux tiers de l'ensemble des diabétiques. Cette fréquence révèle, d'une part la méconnaissance des signes du diabète et, d'autre part le retard fréquent à consulter dans un contexte de faible niveau de couverture sanitaire où les guérisseurs traditionnels sont parfois le premier ou seul recours auxquels font appel les populations en Afrique sub-Saharienne. La Fédération internationale du diabète (IDF) estime à 50% la proportion de diabète méconnu en Afrique [13]. De plus, la prévalence élevée des personnes ayant une hyperglycémie modérée à jeun fait craindre une augmentation du nombre de diabétiques dans les années à venir si l'on tient compte des projections de l'IDF qui estime que 10 à 40% des hyperglycémies modérées à jeun évolueront dans les 5-10 ans vers un diabète. Tout cela prouve que le milieu rural n'est pas épargné par la progression prévue du diabète dans les pays en voie de développement [2, 9, 11, 13]. C'est pourquoi, au Burkina Faso les formations à l'endroit des médecins généralistes et des infirmiers/infirmières des différentes régions afin de décentraliser la prise en charge doivent être poursuivies. Aussi, une place importante doit être accordée aux activités de prévention dans les paquets d'activités des formations sanitaires et du monde communautaire associatif ou leaders à travers des activités de campagne de sensibilisation et de promotion du dépistage. Le dépistage et la prise en charge précoce vont contribuer fortement à réduire le fardeau du diabète en milieu rural.

## Conclusion

Cette étude montre que la prévalence du diabète (connu et méconnu) en milieu rural au Burkina Faso est non négligeable. Les facteurs associés à ce diabète sont l'âge de plus 40 ans, la profession et la surcharge pondérable. L'étude a permis de relever des données en milieu rural concernant l'obésité, le diabète et l'HTA qui sont directement liés à la morbi-mortalité cardiovasculaire. Le coût social de ces maladies doit amener les décideurs politiques à planifier des mesures préventives dans une véritable démarche de santé publique.

## Remerciements

Nous remercions l'Association burkinabè pour la promotion et l'intégration de la jeunesse (ABPIJ), l'Association pour le développement du Zondoma et l'ONG santé-diabète pour leur contribution à la réalisation de la campagne de dépistage du diabète.

## Conflits D'intérêts

Les auteurs ne déclarent aucun conflit d'intérêts.
